# Effects of Incorporation of Jackfruit Rind Powder on Chemical and Functional Properties of Bread

**DOI:** 10.21315/tlsr2018.29.1.8

**Published:** 2018-03-02

**Authors:** Reza Felli, Tajul Aris Yang, Wan Nadiah Wan Abdullah, Wahidu Zzaman

**Affiliations:** 1 Food Technology Division, School of Industrial Technology, Universiti Sains Malaysia, 11800 USM Pulau Pinang, Malaysia; 2 Bioprocess Technology, School of Industrial Technology, Universiti Sains Malaysia, 11800 USM Pulau Pinang, Malaysia; 3 Department of Food Engineering and Tea Technology, Shahjalal University of Science and Technology, Sylhet 3114, Bangladesh

**Keywords:** Jackfruit Rind, Functional Food, Dietary Fiber, Bread, Oil Holding Capacity, Water Holding Capacity

## Abstract

Nowadays, there is a rising interest towards consuming health beneficial food products. Bread–as one of the most popular food products–could be improved to ‘healthy bread’ by addition of ingredients high in protein, dietary fiber and low in calorie. Incorporating Jackfruit rind powder (JRP) as a by-product rich in dietary fiber in bread, could not only provide health beneficial bread products, but also lead to develop an environmental friendly technology by solving the problem of waste disposal of residues. In this study, addition of jackfruit rind powder (JRP) as a high dietary fiber and functional ingredient in bread was examined. The results showed that incorporation of JRP in bread improved functional properties of flour such as Oil Holding Capacity (OHC), Water Holding Capacity (WHC) and pasting properties. Addition of 5%, 10% and 15% of JRP in wheat flour caused significantly (p < 0.05) higher insoluble, soluble and total dietary fiber in flour and bread products. Results from proximate composition indicated that all breads substituted with JRP, contained significantly (p < 0.05) higher fiber, moisture and fat. Obtained results confirmed that the JRP has great potential in development of functional foods especially functional bread products.

## INTRODUCTION

Bread is a staple food prepared from a dough of flour and water, usually by baking. Throughout recorded history it has been popular around the world and is one of the oldest artificial foods, having been of importance since the dawn of agriculture. This acceptance can be ascribed to its distinctive nutritional features related with sensorial and textural properties. Additionally, it is quite easy to prepare and store while it has a reasonably lower price than other stable foods. (Giannou *et al.* 2003). Now a days, consumers are very concise towards healthy foods and they want food with high fibers regarding their health benefits. Bread normally consider as a healthy food because it contains higher dietary fiber and providing lower calorie (Hsu *et al.* 2004; Varastegani *et al.* 2015). Jackfruit seeds are enclosed in a white aril surrounding a thin brown spermoderm which shelters the fleshy white cotyledons. Mostly the seeds are discarded, except sometimes they are roasted or boiled for consumption. Studied described some physico-chemical and rheological properties of the flour and isolated starch from a local jackfruit variety and its partial replacement of wheat flour in white bread (Rengsutthi & Charoenrein 2011). Bread made of composite flours consider as blends wheat after mixture of two or more flours to produce snack foods, leavened breads, unleavened baked, semibaked and porridges products. Blended or composite flours has become popular because of two main reason namely nutritional and economic benefits (McWatter *et al.* 2004; Santiago *et al.* 2015). It might be beneficial to use conposite flour if available local crops resources is utilized that will improve economic condition and accordingly to cut imports pf wheat products (Khalil *et al.* 2000; Murayama *et al.* 2015). The use of Jackfruit rind powder in the manufacture of value-added food products such as breads may profit to consumers and industries.

Most of the earlier studies directed to the utilization of composite flour to make different types of bread were keen to defining the outcome of flours and their proportion to produce quality breads (Feili *et al.* 2013; Khalil *et al.* 2000). The composite flour is prepared using two or more other crops without or with wheat or maize flour. Generally, loaf volume and sensory quality such as texture, appreance and flavour decreased if wheat substitution increased with non-wheat flour (Dhingra & Jood 2004). Khalil *et al.* (2000) stated specifically that the acceptability of a fresh loaf subject to the origin of flour can be achieved by mixing of 30% of cassava flour into wheat flour. Correspondingly Feili *et al.* (2013) reported that the physical properties and sensorial evaluation of bread incorporated with 5% jackfruit rind powder revealed the best quality as consumer acceptance and health benefial bread. The jackfruit rind powder (JRP) can be made using jackfruit ring that presently treated as a by-products of industry handled jackfruit. Upgrading the utilisation of jackfruit by-products from animal feedstuff to functional diet will be of great advantage to the consumer and country. Incorporation of Jackfruit Rind powder (JRP) to make a bakery products might be treated as a source of dietary fiber, value added food, income generation and health benefits for the communities. Moreover the use of Jackfruit rind powder (JRP) is also an environment friendly sustainable technology since it could resolve the solid waste disposal delinquent of residues. Therefore, the aim of the present research work was to utilise the jackfruit rind as dietary fiber ingredients in bread and to determine the chemical and functional properties of the prepared bread.

## MATERIALS AND METHODS

### Preparation of Jackfruit Rind Powder

Jackfruit rind was undergone through several processing steps prior to produce dried jackfruit rind powder. Preparation of jackfruit rind was accomplished according to the technique stated by Feili *et al.* (2013). Jackfruit rinds were separated from the rest of the fruit and were washed with deionised water. After soaking (for 10 min) in boiling water and rinsing the ring were seeped for 10 min in a boiling solution sodium bisulfite (0.1% w/w) and then rinsed. Another soaking step by using boiling solution (sodium bicarbonate) for 15 min were conducted and jackfruit rind pieces were rinsed three times. Then, convection dryer (AFOS Mini Kiln, England) was used to dry them at 50°C for 24 h. The dried samples were powdered using a miller and further sifted over a 355-μm mesh screen. The resulted jackfruit rind powder were preserved in containers (airtight plastic) and kept at 4°C in a fridge prior to use.

### Bread Preparation

Jack fruit ring power (JRP) at the proportion of 5%, 10% and 15% were used with wheat flour to prepare breads as bread substituted 5% JRP (B5JRP), bread substituted with 10% JRP (B10JRP) and bread substituted with 15% JRP (B15JRP), respectively. For spong formation, 50 g wheat flour (WF), 15 g of sugar, 7.6 g instant yeast were applied in 100 mL of water. The ingredients, wheat flour (350 g), milk powder (16 g), sugar (20 g), salt (5 g), improver (9 g), shortening (28 g) and water were utilised for bread making according to the method described by Farinograph (AOAC 2005).

### Analysis of Proximate Composition

The proximate analysis of the breads was undertaken according to the standard method of AOAC (2005). The crude protein, crud fat, crude fibre, ash, carbohydrate (by difference), moisture and calorie were determined for the prepared samples. The analysis were done triplicate and the results were calculated and expressed as gram per 100 gram of dry matter basis.

### Analysis of Total, Soluble and insoluble dietary fibre

The total, sobulble and insoluble dietary fiber of the prepared samples was measured by the AOAC (2005). The percentage of insoluble, soluble and total dietary fibers were determined using the equation mentioned below as Equations 1, 2 and 3 respectively.

(1) $$\%\text{IDF}=\text{R Sample-P-A-B}/{\text{SW}}^{*}100$$

(2) $$\%\text{SDF}=\text{R Sample-P-A-B}/{\text{SW}}^{*}100$$

(3) %TDF=%IDF+% SDF

Where IDF, denotes for Insoluble Dietary Fiber; SDF, denotes for Soluble Dietary Fiber; TDF, symbolises for Total Dietary Fiber; R, denotes for weight (g) of average residue; P, denotes for weight (g) of average protein; A, denotes for weight (g) of average ash, W_1_, represents initial weight of pre dried crucible and celite (g), W_2_, weight of crucible plus celite plus sample after ashing (g), residue weight.

### Analysis of Total, resistant and digestible starch determination

The total starch was determined according to the method stated by Goni *et al.* (1997). The absorbance of the samples standard and blank was read at 500 nm using a UV-VIS spectrophotometer (Shimadzu-USA). The total, resistant and digestible starch resistant starch (RS) was measured as shown in the Equation 4.

(4) % Digestible Starch=Total Starch (%)-Resistant Starch (%)

### Analysis of Water Holding Capacity (WHC) and Oil Holding Capacity (OHC)

The water and oil holding capacity were determined according to the method stated by Chau and Huang (2003) following the Equation 5 and 6 metioned below.

(5) $$\text{WHC}=({\text{W}}_{3}-{\text{W}}_{2})-{\text{W}}_{1}/{\text{W}}_{1}$$

(6) $$\text{OHC}=({\text{W}}_{6}-{\text{W}}_{5})-{\text{W}}_{4}/{\text{W}}_{4}$$

Here, W_1_ and W_4,_ denotes weight of samples (g); W_2_ and W_5,_ denotes weight of pre-dried centrifuged tube (g); W_3_ and W_6_ denotes weight of sample and centrifuge tube.

### Analysis of Pasting Properties

The pasting properties were measured according to the method of AOAC (2005). For determination of pasting properties, a rapid visco analyser (Newport Scientific Pty Ltd, Australia) were used. Approximately 3g of smples were taken and dispersed in aluminum canister containing distilled water. For moisture correction the following equation were applied.

(7) $$\begin{array}{l} {\text{M}}_{1}=(100-14)\times {\text{M}}_{2}/100-{\text{W}}_{1}\\ {\text{W}}_{1}=25.00+({\text{M}}_{2}-{\text{M}}_{1}) \end{array}$$

Where M_1,_ denotes the weight of corrected sample (g); M_2,_ denotes weight of sample (3 g), W_1_, denotes actual moisture content of sample (%); W_2,_ denotes corrected water volume (mL), and 25 mL is the volume to suspend 3 g of sample. The suspension of flour-water was kept at 50°C for 1 min and heated up to 95°C and kept for 10 min. The parameters of starch viscosity such as Pasting Viscosity (PV), Pasting Temperature (PT), Final Viscosity (FV), Break down (BD) and Set Back (SB) were measured and the results were expressed as Rapid Visco Units (RVU).

### Statistical Analysis

All samples were examined in triplicates and the results were analysed by using SPSS 18.0 Software (SPSS Inc., Chicago, USA). Furthermore, significant differences between the mean values were determined by using the analysis of variance (ANOVA) and Duncan’s multiple range tests were expressed at a significance level of *P* < 0.05.

## RESULTS AND DISCUSSION

### Soluble, Insoluble and Total Dietary Fiber

**Figure 1:**
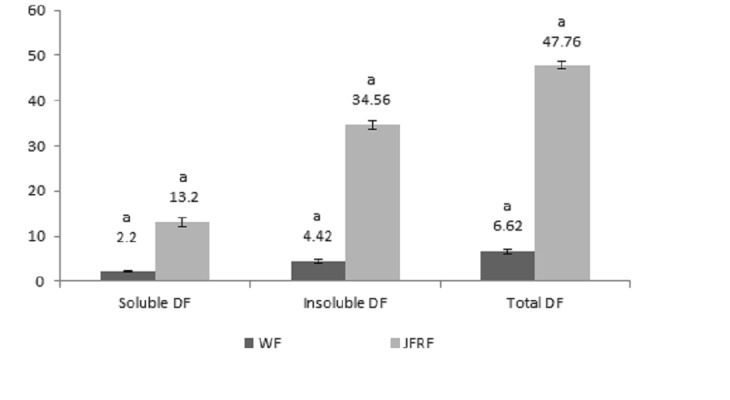
Dietary fiber percentage of jack fruit ring (JRP) vs. Commercial wheat flour (WF).

The percentage of soluble, insoluble and total dietary fiber contents are shown in Figure 1. Total dietary fiber comprises soluble dietary fibers (SDF) and insoluble dietary fibers (ISD) respectively. The result showed that jack fruit ring powder (JRP) was significantly (*p* < 0.05) higher amounts of soluble, insoluble dietary fiber and consequently, total dietary fiber were compared to commercial wheat flour (WF). The most dominant fiber was insoluble either in JRP (34.56%) or WF (4.42%) presented in Figure 1. The similar trend found for total dietary fiber (TDF) percentage in JRP and WF. The study observed that the TDF contents in JRP (47.76%) was significantly (*p* < 0.05) higher as compared to WF (6.62%). According to Abdul-Hamid *et al.* (2000) reported that the TDF contents in JRP was higher significantly than that of oat bran (26.40%). The research stated by Chen *et al*. (1998) also found significantly higher amount of TDF in JRP (47.76%) than rice bran (27.4%) samples. The result obtained from our study found JRP are rich soucr that can be applied in bakery products as a fiber rice value added product.

### Digestible, Resistant and Total Starch

**Figure 2:**
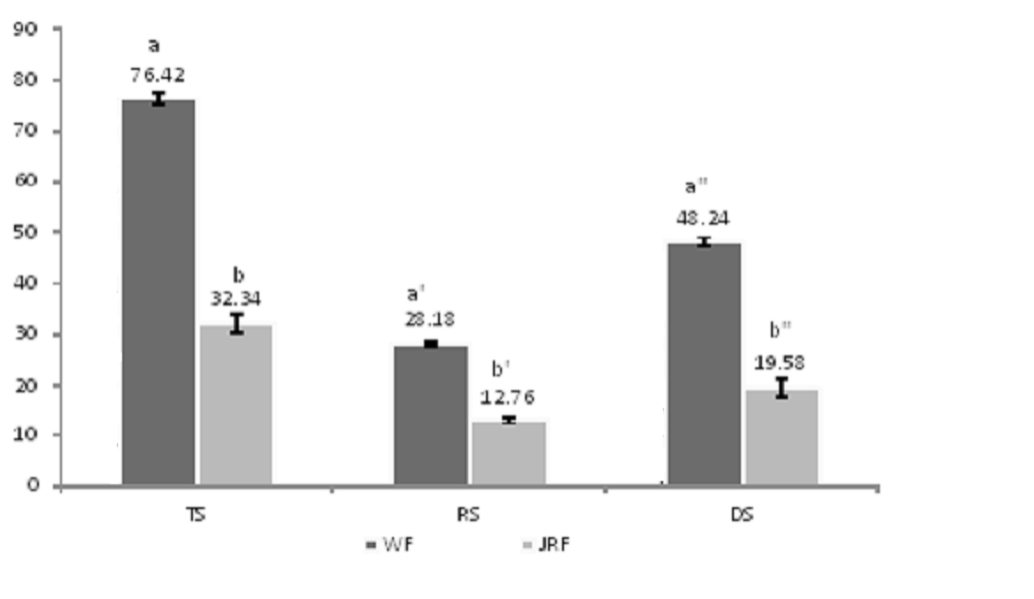
Total and resistant starch percentage of jackfruit rind powder (JRP) vs Commercial wheat flour (WF).

The results of digestible starch (DS), resistant starch (RS) and total starch (TS) contents of WF and JRP are presented in Figure 2. From the results obtained in this study showed significantly (*P* < 0.05) lower TS value (32.34%) in jack fruit ring powder (JRP) as compared to wheat flour (WF) samples (76.42%). Similarly the RS content (12.76%) in JRP was also significantly (*p* < 0.05) lower than WF (28.18%). According to Goni *et al.* (1996) suggested that the resistant starch content (12.76%) in JRP and (28.18%) in WF categorised as high resistant starch content products. The resistant starch help to pass indigestible foods from human intestine through the colon. It acts as a dietary fiber and reduce transit time during digestion. Food enegy from ingested foods may be also influenced by dietary fiber termed as resistant starch with fermentation by bacterial growth in colon (Goni *et al.* 1997). The amount of resistant starch can be increased by heat-moisture treatment during processing.

### Oil Holding Capacity (OHC) and Water Holding Capacity (WHC)

**Figure 3:**
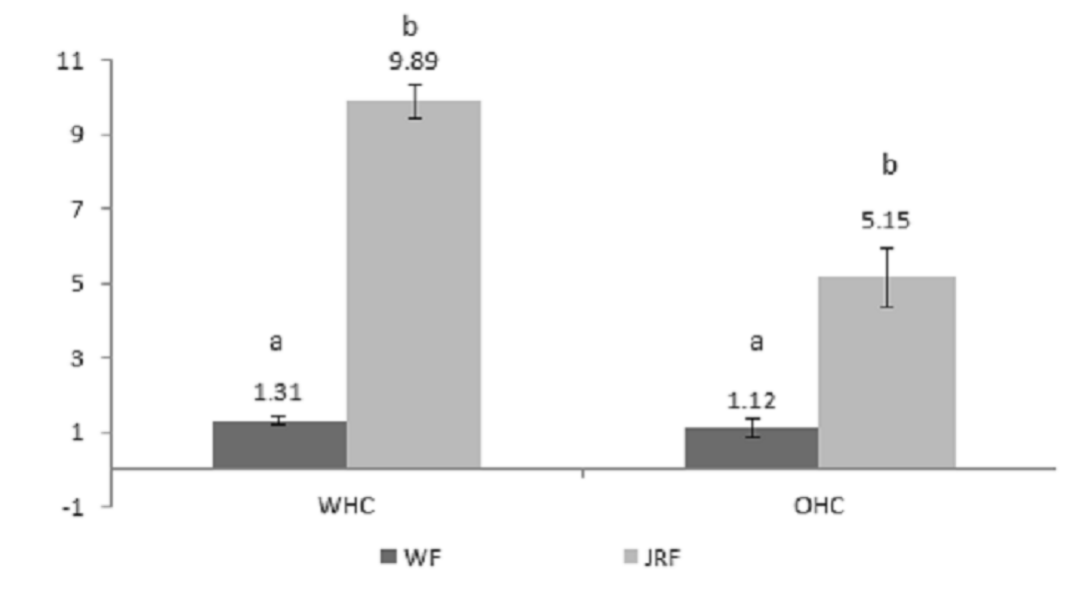
Water holding capacity (WHC) and Oil holding capacity (OHC) of jackfruit rind powder (JRP) vs. Commercial wheat flour (WF).

Oil Holding Capacity (OHC) and Water Holding Capacity (WHC) of JRP were significantly (*p* < 0.05) higher than those of commercial wheat flour (WF) is shown in Figure 3. The both parameters, oil holding and water holding capacity have a vital role in the functional properties and final product quality. The water holding capacity (WHC) in the study found 1.31 and 9.89 g of water/g of dry matter in WC and JRP respectively. The water holding capacity (WHC) in jackfruit ring power (JRP) was higher as compared to oat bran (2.10 g), rice bran (4.89 g), wheat bran (5.03 g) and soy flour (6.75 g) (Abdul-Hamid & Luan 2000; Heywood *et al.* 2002). From the result observed, the jackfruit ring powder (JRP) is proficient of binding additional water than soy, WF, rice bran, oat bran and flour. Additional boiling during processing may slightly affect the WHC in jackfruit ring powder. In addition, oil holding capacity (OHC) of JRP found 5.15 g of oil/g of dry matter which was about five times higher as compared to the wheat flour (1.12 g). This variation of oil holding capacity between whaet flour and jackfruit ring powder could be explicated by their change in chemical and physical properties. Therefore the variation in amount of insoluble fiber could be an vital factor in oil holding capacity of WF and JRP. The important of OHC is crucial for the use of flour in cooked or fried products by developing oil holding capacity and providing the cooking yield in food industry. Alternatively, the dimension of fiber elements can also influences OHC and WHC of food products. Therefore, JRP appears quite appropriate for the use in food industries as a soothing material to upturn and steady the cooking yield (Thebaudin *et al.* 1997).

### Pasting Properties

The result of pasting properties with different proportion combinations of JRP and WF (C: control; wheat flour), 5JRP: wheat flour blended with 5% JRP, 10JRP: wheat flour blended with 10% JRP, 15JRP: Wheat flour blended with15% JRP) were evaluated and the findings are presented in Table 1. The results of pasting properties of WF and JRP exhibited significant variation (*P* < 0.05) in most of the pasting characteristics. The pasting temperature, the temperature at which initial swelling of starch granules takes place when suspended in wate, is very important to evaluate the properties of flour. (Kaur *et al*. 2011). Thus, it can indicate the firmness of other constituents in the formulation of the sample. The pasting temperature of the samples were not significantly different (*P* > 0.05). Therefore, the different sample and control showed similar resistance to rapture and puffiness (Miao *et al.* 2009). The highest viscosity value provides an indication of swelling properties and water-binding capacity of the flour starch. The starch granual twitch to swell in the presence of water on heating. Meanwhile some components like amylose and amylopectin diffuse out which cause particle dispersion in a continuous phase (Thebaudin *et al.* 1997). Therefore, the starch viscosity increases until its maximum value. Lower peak viscosity value observed in 15JRP compared to other samples implied that there are higher swelling powers in sample formulated with higher JRP. Our results (Table 1) indicated that pick viscosity value for 5JRP and 10JRP were higher than 15JRP. Meanwhile, C had the highest pick viscosity value. The ability of a sample to withstand the shear stress and heating is known as Breakdown Viscosity value (BV). The result of breakdown viscosity value of the three samples i.e. 5JRP, 10JRP, and 15JRP were significantly (*P* < 0.05) different from C in Table 1. Nevertheless, there was no significant difference (*P* > 0.05) among the samples. Their lower breakdown viscosity value in comparison with control showed that there are greater shear forces in bread samples containing JRP compared to those without JRP. Moreover, it suggests that there are robust bonding forces inside the structure of starch containing JRP during the heating in bread samples. Final Viscosity (FV) specifies the stability and quality of cooled cooked paste under low shear. The BC value was significantly higher (*P* < 0.05) FV value than other bread containing JRP samples are shown in Table 1. The FV value decreased (5JRP > 10JRP = 15JRP) when increasing the volume of JRP substitution. Consequently, 5JRP is more proficient of making gel after cooling and cooking as compared to 10JRP and 15JRP. Hence it could be applied in food product development e.g. puddings which are necessary to condense and form gel. The degree of the variance between final viscosity and peak viscosity is recognised as setback. The value can display the steadiness of the gel and comprises retrogradation of starch particles (Miao *et al.* 2009). According to our outcomes (Table 1), there was a significant difference (*P* < 0.05) between the control (BC) and samples and. When the amount of JRP increased it led to a decline in setback. It exhibited that JRP comprises less amylopectin and amylose than that of WF. Nimsung *et al.* (2007) also stated that products comprising starch with lesser level of amylose would undertake a compact retrogradation than those with high amount of amylose starch.

### Proximate Composition

Results of proximate composition of different bread samples with different level of JRP (on dry basis) are shown in Table 2. The moisture content of the samples were significantly (*P* < 0.05) higher when the level of JRP in bread samples are increased. This could be ascribed to high fiber content of jackfruit ring powder. According to Rodriguez *et al.* (2006) described, fruit fibers naturally have high level of moisture with some small amounts of vascular tissues. In addition to increase in moisture, crude fat and crude fiber also showed gradual significant increase (*P* < 0.05) by addition of JRP to bread samples. According to Feili *et al.* (2013) results of proximate composition in JRP exhibited higher content of crude fiber (11.32 g/100 g of dry matter), crude fat (0.82 g/100 g of dry matter) and ash (5.91g/100 g of dry matter) as compared to wheat flour. The study found a lower level of moisture (9.43g/100 g of dry matter), and crude protein (4.52 g/100 g of dry matter) in JRP. Therefore, higher fiber and fat content in bread made from different proportion of JRP could be due to higher content of crude fat and crude fiber in JRP. Though, carbohydrate, crude protein and ash exhibited by the addition JRP in the formulation of the bread products. Normally, WF comprises higher quantity of carbohydrate, protein and ash content than JRP (Feili *et al.* 2013). The calorie value also decreased significantly (*P* < 0.05) by the addition of JRP into the formulation of the bread products. This could be explicated by significantly lower amount of carbohydrate and protein in JRP, which provides it a low-calorie. Thus it could be a potential bakery ingredients to produce fiber rich bread.

### Dietary Fiber in Bread Samples

The results of total, soluble and insoluble dietary fiber (TDF, SDF and IDF) contents are presented in Figure 4. According to the results obtained from this study, there were significant (*p* < 0.05) differences among the TDF, SDF and IDF content. The major portion was insoluble dietary fiber. By addition of JRP into the formulation of bread samples, the TDF significantly (*P* < 0.05) increased from 3.87% in BC to 11.39% in B15JRP (Figure 4). Likewise, the IDF content significantly increased (*P* < 0.05) in samples containing JRP. There was no significant difference (*P* > 0.05) between SDF of bread substituted with 5% JRP (B5JRP) and control bread (BC). However, addition of JRP to 10% and15% affected significant difference (*P* < 0.05) in their content of IDF. The reason of increase in TDF could be due to related increase in the SDF and IDF content of bread products replaced by JRP. In terms of healthiness, SDF and IDF both are important for the biological activities in human. Water absorption capacity and intestinal regulation function is more affected by the IDF, though controlling diabetic and lowering cholesterol are more ascribed to SDF (Prosky *et al.* 1992). According to Official Journal of the European Union (2006) reported that breads developed with JRP might be well-known as high-fiber when they added more than 6g dietary fiber/100g food. The current recommendation for fiber intakes for adults is ranged from 25 g to 30 g per day with an IDF/SDF ratio of 3:1 (Borderias *et al*. 2005). In the present study, the ratio of IDF/SDF found 2.14:1, 2.73:1, 2.95:1 and 2.98:1 in BC, B15JRP, B5JRP and B10JRP respectively. Therefore, breads with added JRP (especially 10%) are possibly a good source of dietary fiber food.

### Total, Resistant and Digestible Starch in Bread Samples

The total starch content of bread samples were 74.13% in B15JRP to 81.04% in BC. After incorporation of JRP in the formulation of bread samples, the TS vale significantly (*P* < 0.05) decreased presented in Table 3. This could be described by the point that TS was lower level in JRP as compared to WF (Figure 2). Subsequently, partial replacement of JRP in BC to make B5JRP, B10JRP and B15 JRP produced lower TS value in the final samples. In contrary to results of TS, resistant starch (RS) of prepared bread samples presented significant increase (*P* < 0.05) by partially replacement of JRP in BC. This increase in RS might be explicated by the proportion of RS: TS in JRP and WF. Since it contained a higher proportion of RS: TS in JRP (39.4%) than that of WF (36.8%), by replacement of JRP in the formulation of bread products, the RS value of bread samples showed significantly (*P* < 0.05) higher. The digestible starch (DS) in bread samples replaced with JRP also shown improved significantly (*P* < 0.05). This might be ascribed to higher fiber and resistant starch in B5JRP, B10JRP and B15JRP. Digestible starch of BC found in this study was lower (67.78) than what was reported (74.2%) by Goni *et al.* (1997). According to Liljeberg *et al.* (1996) stated that the RS value may be affected by altering the temperature and time of baking, and it would upsurge by altering the conventional baking method to low temperature for longer time period during baking.

## CONCLUSION

Results acquired from functional and chemical analysis directed that JRP obtained from treated rind of jackfruit showed the potential ingrdient to be applied in bread in a partial ratio (5%) as health advantageous composite flour. This powder exhibited a rich source of nutritive fiber food which can improve the functional characteristics of flour and bread. The breads containing JRP were significantly (*P* < 0.05) affected the functional and chemical properties of the products. Results obtained from proximate analysis specified that all breads replaced with JRP, ensured significantly (*P* < 0.05) higher fiber content. The functional properties of bread made from flour partially replaced with JRP such as Oil Holding Capacity (OHC), Water Holding Capacity (WHC) and pasting properties upgraded significantly (*P* < 0.05). From the abovemetioned findings it is concluded that JRP may be applied as a novel ingredient to produce high quality bakery products with an optimum ratio specially bread products. Further works on biochemical and antioxidant properties with different cultivar should be carried out to produce fruit based high fiber bread.

## Figures and Tables

**Table 1:** Pasting properties of different type of flour samples.

Samples	PT(°C)	PV (RVU)	BD (RVU)	FV	Setback(RVU)
C	65.83±0.34^a^	184.26±10.03^a^	87.31±1.24^a^	205.34±3.04^a^	102.11±2.08^a^
5JRP	66.45±0.65^a^	167.51±5.34^b^	79.41±3.42^b^	193.62±0.16^b^	96.54±0.23^b^
10JRP	66.67±0.49^a^	163.43±6.20^b^	74.35±1.03^b^	189.08±0.38^c^	89.03±0.42^c^
15JRP	65.53±1.95^a^	154.38±2.32^c^	75.65±0.34^b^	188.64±0.53^c^	88.46±0.48^c^

* Data are average values of triplicate ± standard deviation.

Values in the same column with different superscripts are statistically significant (*P* < 0.05). PT: Pasting Temperature, PV: Peak Viscosity, BD: Breakdown, FV: Final Viscosity, RVU: Rapid Visco Units, C: control (wheat flour), 5JRP: wheat flour blended with 5% JRP, 10JRP: wheat flour blended with 10% JRP, 15JRP: Wheat flour blended with15% JRP

**Table 2:** Proximate composition of different type of bread samples.

% Composition	BC	B5JRP	B10JRP	B15JRP
Moisture	40.12±0.52^d^	42.24±0.32^c^	43.36±0.11^b^	44.94±0.23^a^
Crude fat	2.21±0.22^c^	2.24±0.42^c^	2.48±0.05^b^	2.63±0.26^a^
Crude protein	14.76±0.76^a^	13.04±0.32^b^	12.57±0.34^b^	11.43±0.14^c^
Ash	3.54±0.23^a^	3.43±0.02^b^	3.21±0.34^b^	3.06±0.04^c^
Crude Fiber	1.15±0.30^d^	3.36±0.46^c^	4.65±0.32^b^	6.02±0.23^a^
Carbohydrate	40.61±0.52^a^	35.69±0.20^b^	33.73±0.19^c^	31.92±0.09^d^
Calorie^e^	221.48± 0.62^a^	215.08±0.24^b^	207.52±0.15^c^	197.07± 0.13^d^

* Data are average value of triplicate ± standard deviation.

Values in the same row with different superscripts are significantly different (p < 0.05).

e (Kcal/100 g of dry matter).

**Table 3:** Total, resistant and digestible starch of different type of bread samples.

Composition	Total starch	Resistant Starch	Digestible starch
BC	81.04±0.53a	13.26±0.14a	67.78±0.36a
B5JRP	78.21±0.21b	13.83±0.06b	64.38±0.27b
B10JRP	75.82±0.34c	14.11±0.16c	61.71±0.29c
B15JRP	74.13±0.03d	14.72±0.32c	59.41±0.08d

* BC: White wheat bread, B5JRP: Bread substituted with 5% JRP, B10JRP: Bread substituted with 10% JRP, B15JRP: Bread substituted with 15% JRP. Values in the same row with different superscripts are significantly different (*P* < 0.05).
